# Symbolic numerical generalization through representational alignment

**Published:** 2025

**Authors:** Anthony Strock, Ruizhe Liu, Rishab Iyer, Percy K. Mistry, Vinod Menon

**Affiliations:** 1 Department of Psychiatry & Behavioral Sciences, Stanford University School of Medicine, Stanford, CA, USA; 2 Department of Neurology & Neurological Sciences, Stanford University School of Medicine, Stanford, CA, USA; 3 Wu Tsai Neurosciences Institute, Stanford University School of Medicine, Stanford, CA, USA

**Keywords:** Emergence of number semantics, Representational alignment, Artificial neural network

## Abstract

The mapping between nonsymbolic quantities and symbolic numbers lays the foundation for mathematical development in children. However, the neural mechanisms underlying this crucial cognitive bridge remain unclear. Here, we investigate the computational principles governing symbolic-nonsymbolic integration using a biologically inspired neural network trained through developmentally inspired stages. Our investigation reveals that generalization from nonsymbolic to symbolic numerical processing emerges specifically when representational alignment forms between these numerical formats. Notably, this alignment appears to be stronger in cross-format comparison-based mapping compared to direct-label-based mapping. Furthermore, we demonstrate that subsequent symbolic specialization creates a representational divergence that impairs nonsymbolic performance while maintaining the ordinal structure of the mapping. These findings highlight representational alignment as a fundamental mechanism in numerical cognition and suggest that targeted cross-format comparison tasks may be particularly effective in improving mathematical learning in children with numerical processing difficulties.

## Introduction

Numbers are fundamental to human cognition and shape how we measure and make sense of our world. In the early stage of learning, children can discriminate and compare quantities in different perceptual domains, such as different numbers of visual objects, sounds, or physical touches (Lipton & Spelke, 2003; Xu & Spelke, 2000). As development progresses, children acquire counting skills, which fundamentally involve mapping quantities to symbolic representations such as number words (Wynn, 1990; Sarnecka & Carey, 2008). The successful formation of these symbolic-nonsymbolic associations proves critical for subsequent mathematical development (Holloway & Ansari, 2009; Schwartz et al., 2021).

A distinctive characteristic of humans’ numerical representations, in both symbolic and nonsymbolic domains, is adherence to Weber’s law (Krueger, 1984; van Oeffelen & Vos, 1982). This psychophysical principle reveals that performance in numerical tasks depends on the ratio between quantities rather than their absolute difference, suggesting similar underlying representational mechanisms for both symbolic and nonsymbolic numbers. Neuroimaging evidence further supports this view, demonstrating that neural representations of symbolic and nonsymbolic numbers show substantial similarities early in development, before diverging as individuals gain expertise with symbolic numbers (Park et al., 2024). This developmental trajectory suggests that the neural number system evolves in stages—initially establishing mappings between nonsymbolic and symbolic representations to facilitate generalization, before refining symbolic representations to enhance performance on symbolic tasks. However, the precise neural mechanisms supporting this mapping process and its role in generalization remain poorly understood.

In this study, we investigate the neural representation of numerical cognition by simulating both symbolic and nonsymbolic number processing in a biologically inspired neural network using comparison tasks. Drawing on developmental trajectories observed in children’s mathematical learning, we implement a three-stage training sequence: (1) nonsymbolic training where the model learns to compare dot arrays, (2) mapping training where connections between symbolic and nonsymbolic representations are established, and (3) symbolic training where the model learns numeral comparisons. Crucially, we compare two distinct mapping approaches—cross-format comparison (where the model compares quantities across different formats) and cross-label mapping (where both formats are associated with the same labels)—to understand how different learning pathways affect representational development and generalization.

Neurophysiological studies have identified ”number neurons”—neurons selectively tuned to specific quantities—in both non-human primates and artificial neural networks (Nasr, Viswanathan, & Nieder, 2019; Kim, Jang, Baek, Song, & Paik, 2021; Mistry, Strock, Liu, Young, & Menon, 2023; Chapalain, Thirion, & Eger, 2024). These specialized neurons emerge even without explicit numerical training when exposed to nonsymbolic stimuli such as dot arrays (Nasr et al., 2019; Kim et al., 2021). Furthermore, research suggests that linear representational structures underlie numerical processing in both humans and artificial networks performing numerical and ordinal comparison tasks (Sheahan, Luyckx, Nelli, Teupe, & Summerfield, 2021; Nelli, Braun, Dumbalska, Saxe, & Summerfield, 2023). Building on these findings, we examine whether mapping between nonsymbolic and symbolic number representations enforces an alignment of their respective neural representations, thereby enabling generalization to symbolic numerical tasks. We also investigate how subsequent explicit symbolic training affects this alignment.

## Methods

### Tasks

#### Dot stimuli (nonsymbolic).

**Figure 1:**
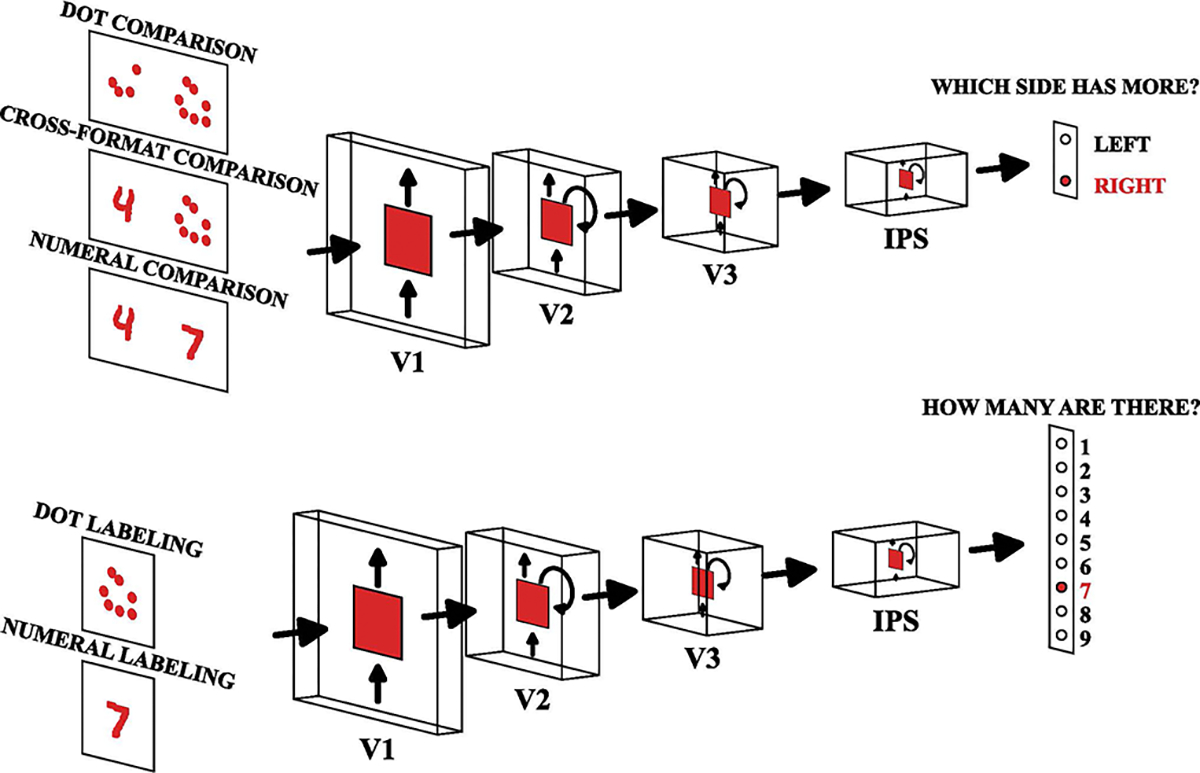
Numerical processing neural network. Illustration of our biologically inspired neural network model processing numerical information. The top panel shows the model performing comparison tasks (determining which side contains more), while the bottom panel shows quantification tasks (determining how many items are present).

**Figure 2:**
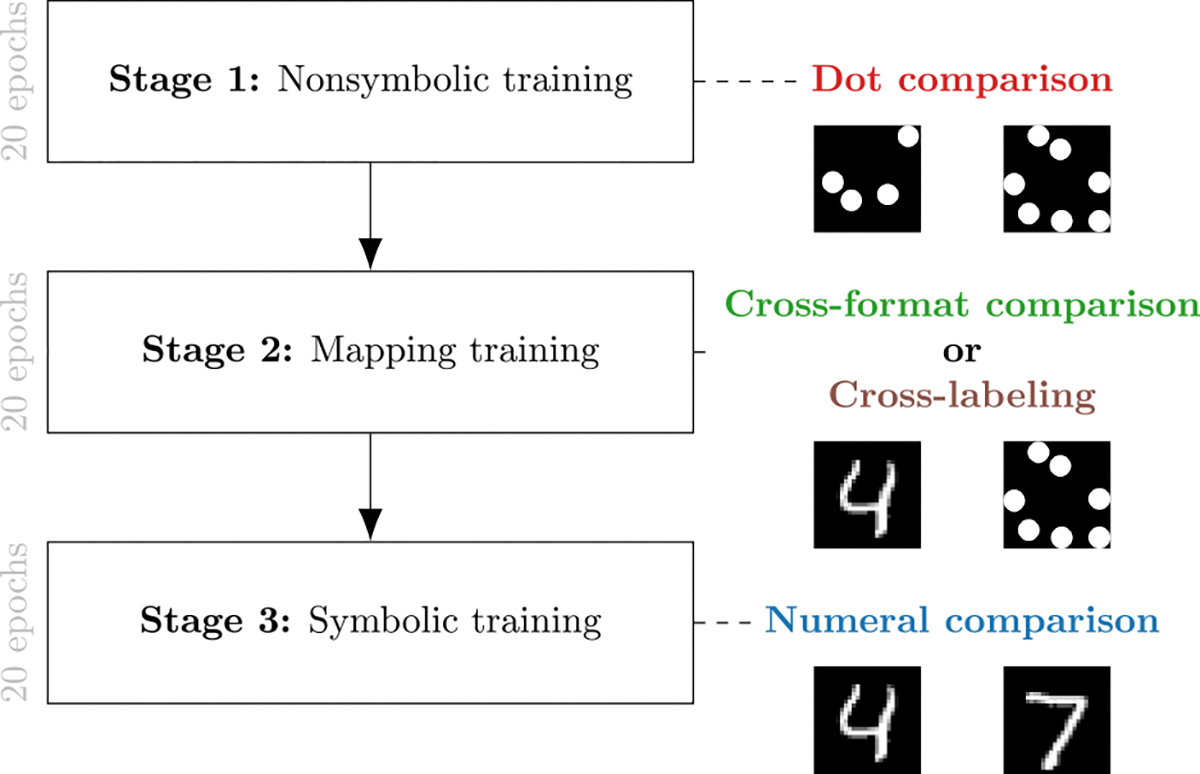
Developmental training progression. Stage 1 (Nonsymbolic training): The model learns to compare dot arrays. Stage 2 (Mapping training): The model learns to connect symbolic and nonsymbolic representations through either cross-format comparison or cross-labeling. Stage 3 (Symbolic training): The model learns numeral comparison. Throughout the paper, dot comparison tasks are represented in red, numeral comparison tasks in blue, cross-format comparison task in green, and cross-labeling task in brown.

Our dataset consisted of stimuli images with 1–9 dots (224 × 224 pixels). All dots in an image were of a single color, but colors were randomly generated across images. All images were generated with a target total area and target convex hull area as parameters. For each numerosity 1–9, we generated 12 images (10 training, 2 testing) each for a set of 50 different parameters, resulting in 9 × 12 × 50 = 5400 images (4500 training, 900 testing). For visualization purposes, the stimuli are shown in white and red (Figure 1), or black and white (Figure 2) in the paper.

#### Numeral stimuli (symbolic).

Our dataset consisted of stimuli images with numerals from 1–9, using a subset of handwritten digits from the MNIST dataset (Deng, 2012). For each numerosity n we sampled the first 500/100 (training/testing) handwritten digits n from the MNIST dataset, to match the dot stimuli sample size. To match the physical size of the dot and numeral stimuli, the MNIST pictures were upsampled from their original size (28 × 28) to (224 × 224) by replicating each pixel uniformly into a constant subregion of size (8 × 8) without applying any additional filtering.

#### Symbolic, nonsymbolic and cross-format comparisons.

Comparison task stimuli consisted of two different numbers between 1 and 9, each represented by a (224 × 224) image. The desired model output was a categorical one-hot coded choice describing which number was higher (i.e. left or right). We used 3 different comparison tasks: (1) nonsymbolic format comparison, (2) symbolic format comparison, and (3) cross-format comparison, where one number was represented as a symbolic (numeral) and the other as a nonsymbolic (dot) image. For each task, we created a training/testing set of 4500/900 comparisons, by uniformly sampling pairs of two different numbers between 1 and 9 for each stimuli.

#### Numerical cross-labeling.

We considered numerical labeling task where an image of nonsymbolic or symbolic numbers had to be associated with a label from 1–9. The input stimulus was a single (224 × 224) image, and the desired output was a categorical one-hot choice representing a number between 1 and 9. We used three different tasks, where learning was based on (1) nonsymbolic stimuli only (4500/900 training/testing images), (2) symbolic stimuli only (4500/900 training/testing images), and (3) both symbolic and nonsymbolic stimuli (9000/1800 training/testing images, merging the datasets from the first two tasks).

### Model

#### Biologically inspired model of the dorsal visual pathway.

Our model is adapted from CORnet-S (Kubilius et al., 2018) to replicate the dorsal visual pathway involved in numerical cognition, including four key layers – visual layers V1, V2, V3 and the intraparietal sulcus (IPS) layer, corresponding to key brain regions involved in numerical information processing from visual stimuli (Skagenholt, Träff, Västfjäll, & Skagerlund, 2018; Castaldi, Piazza, Dehaene, Vignaud, & Eger, 2019). The output dimension of the last linear decoder of CORnet-S was changed based on task requirements (2 classes for the comparison task and 9 classes for the quantification tasks). For additional details, see (Mistry et al., 2023).

#### Developmentally inspired training.

To mimic developmental learning and how children are gradually exposed to numerical comparisons, we trained our model in three stages (Figure 2): (1) nonsymbolic comparison training for 20 epochs, (2) nonsymbolic-symbolic mapping training for 20 epochs, and finally (3) symbolic comparison training for 20 epochs. We compared two ways of performing the intermediate mapping training, using: (1) cross-format comparison, and (2) cross-label mapping. We observed partial forgetting of dot comparison when the mapping stage involved cross-labeling, but not when we used cross-format comparison. We hence interleaved cross-label mapping training with nonsymbolic dot comparison (but not with symbolic comparison).

### Behavioral analysis

#### Accuracy and pair accuracy.

Unless specified otherwise, accuracy refers to the testing accuracy for the comparison task, measured across all stimuli. We refer to **accuracy** as the accuracy across all stimuli. We refer to the (*n, p*) pair accuracy as the accuracy across all the stimuli for which the underlying pair of numbers is (*n, p*).

#### Generalized cross-format and symbolic pairs.

We define generalized symbolic (resp. cross-format) pairs as the pairs of numbers (*n, p*) such that the symbolic (resp. cross-format) (*n, p*) pair accuracy at the end of the mapping (resp. nonsymbolic) training stage is above 95%.

#### Forgotten nonsymbolic pairs.

Similarly, we define the forgotten nonsymbolic pairs as the pairs of numbers (*n, p*) such that the nonsymbolic (*n, p*) pair accuracy is above 95% at the end of the mapping symbolic training stage but below 55% at the end of the symbolic training stage.

### Representational analysis

#### Neural representational similarity.

To compute neural representational similarity (NRS) (Kriegeskorte, 2008), we examined the neural response of the model in the quantification tasks. In other words, we provided (224 × 224) pictures containing either a single dot array or a single numeral as stimuli to the model and examined its responses. Specifically, for each number n and format f∈s,ns (i.e. symbolic or nonsymbolic), we computed the average response ${\overset{‾}{x}}_{n,f}$ of the model’s IPS layer across pictures in the test datasets. We then computed the similarity between two average responses ${\overset{‾}{x}}_{n,f}$ and ${\overset{‾}{x}}_{n^{\prime },f^{\prime }}$ as $1-\frac{{\left\|{\overset{‾}{x}}_{n,f}-{\overset{‾}{x}}_{n^{\prime },f^{\prime }}\right\|}_{2}}{\underset{p,p^{\prime },g,g^{\prime }}{\max}{\left\|{\overset{‾}{x}}_{p,g}-{\overset{‾}{x}}_{p^{\prime },g^{\prime }}\right\|}_{2}}$.

#### Multi-dimensional scaling.

We computed a 3-dimensional Multi-Dimensional Scaling (MDS) (Mead, 1992) of the average responses of the model’s IPS layer ${\overset{‾}{x}}_{n,f}$ for each number n and format f, by precomputing the norm-2 distance between averages ${\left\|v-{\overset{‾}{x}}_{n^{\prime },f^{\prime }}\right\|}_{2}$. Since the MDS space is defined up to an orthonormal transformation, we rotated the MDS representation such that the first dimension of a number n is best aligned with n. The representation on the first rotated dimension is denoted as $r_{n,f}$.

#### Representational alignment.

To compute the alignment between symbolic and nonsymbolic representation, we measured: (1) the correlation between $r_{n,s}$ and $r_{n,ns}$ across numbers n, and (2) the normalized average distance $\frac{1}{9}\sum_{n=1}^{9}\;\frac{\left|r_{n,s}-r_{n,ns}\right|}{\left|r_{9,ns}-r_{1,ns}\right|}$. For quantitative analysis of alignment, we used only the first dimension of rotated MDS, but for visualization purposes, we displayed the two first dimensions.

### Code availability

Code will be made available on GitHub upon publication at https://github.com/scsnl/Strock_CogSci_2025.

## Results

### Generalization from cross-format comparison

Our first objective was to determine how and when generalization to symbolic comparison emerges during developmentally-inspired training. We examined performance through a three-stage training process that mimics numerical development in children. We designed the cross-format comparison mapping to simulate how children might establish numerical meaning by directly comparing quantities across formats, such as when they compare a set of objects to a written numeral. This approach mirrors natural learning situations where children must determine which of two quantities (one symbolic, one nonsymbolic) is larger, potentially establishing more integrated magnitude representations through relational judgments.

#### Initial nonsymbolic comparison learning.

**Figure 3:**
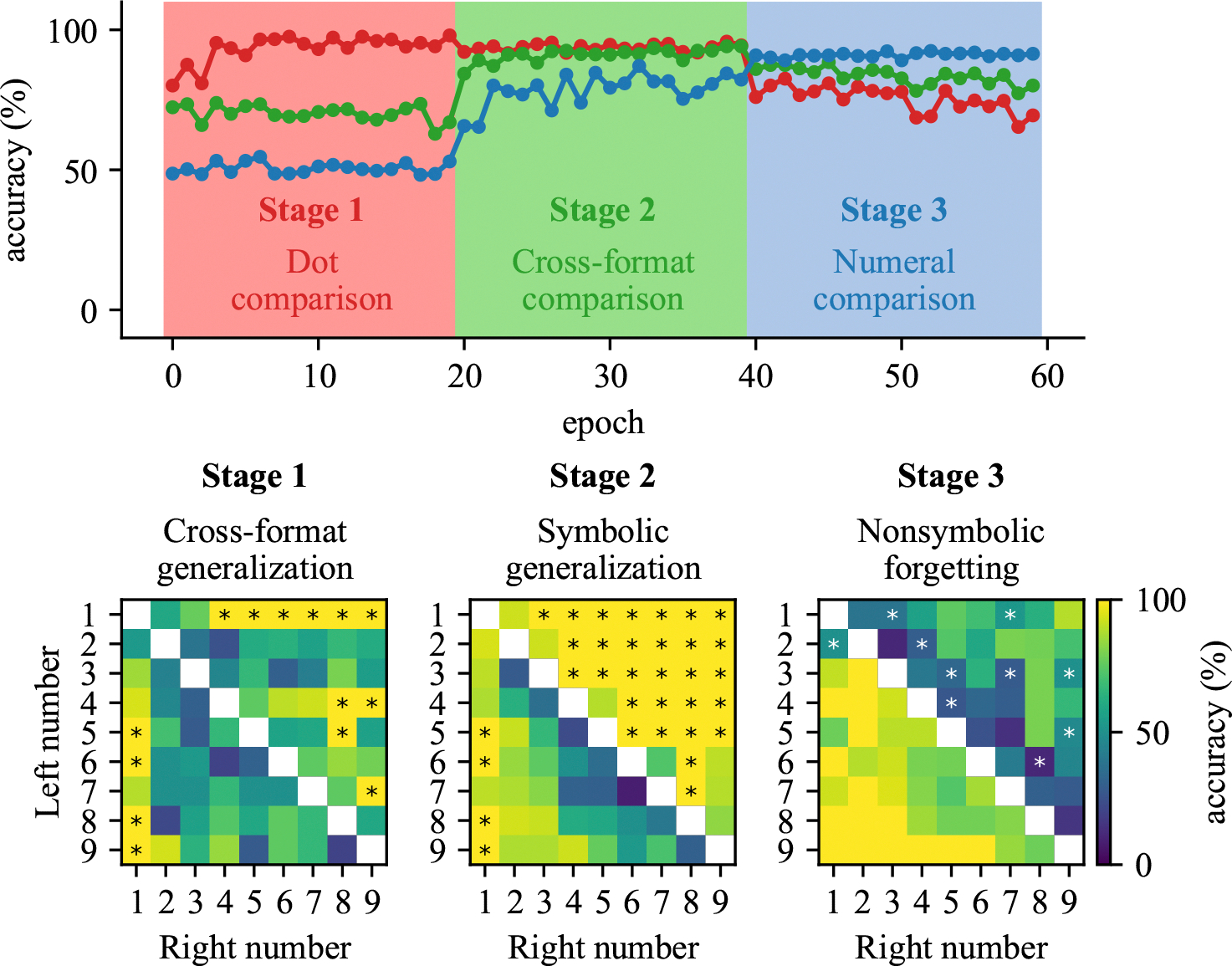
Performance of model developmentally trained with a cross-format comparison training. (Top) Solid line represents the accuracy in test on the dot, cross-format, or numeral comparison task. The color of shaded area represents the new task that was trained. (Bottom) Pairs that are generalized and forgotten across training, from generalized cross-format pair in Stage 1, generalized symbolic pair in Stage 2, to forgotten nonsymbolic pair in Stage 3.

In Stage 1, nonsymbolic training resulted in high levels of performance on nonsymbolic comparisons, as expected, but also partial generalization to cross-format comparisons, achieving accuracy above 65% compared to the 50% chance level (Figure 3, top panel). The strongest early generalization was found for number pairs containing either dot or numeral 1 (Figure 3, bottom panel, left). When 1 appears in a comparison, the outcome becomes deterministic - when 1 dot appears on the left, the right side always has more, and vice versa. Interestingly, this generalization occurred not only for dot 1 but also for numeral 1, suggesting early cross-format integration for this specific number.

#### Robust generalization to symbolic comparison.

In Stage 2, we examined the effects of cross-format mapping. When mapping was established through cross-format comparison (direct comparison between dot arrays and numerals), the model achieved substantial generalization to purely symbolic comparison tasks, reaching 82% accuracy after mapping training (Figure 3, top panel). This generalization showed a distinct directional bias, with higher accuracy for pairs where the right number exceeded the left number (Figure 3, bottom panel, middle). This asymmetry resembles the SNARC (Spatial-Numerical Association of Response Codes) (Dehaene, Bossini, & Giraux, 1993) effect in human cognition, suggesting the emergence of a directional mental number line.

#### Neural representational development.

**Figure 4:**
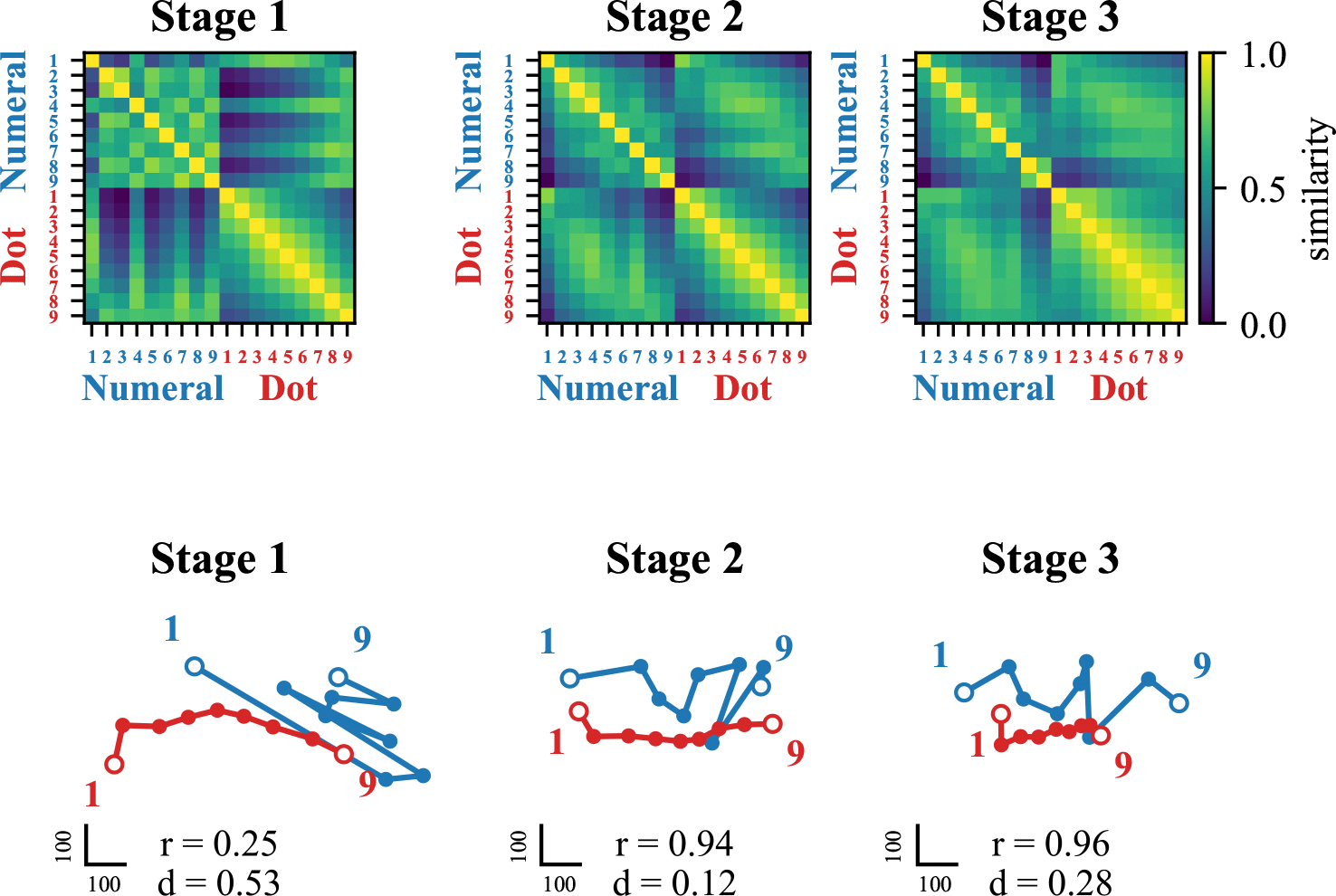
Progressive alignment of symbolic and nonsymbolic number representation for model developmentally trained with a cross-comparison training. (Top) Neural representational similarity (NRS) matrices revealing distance effects (where numbers closer in value have more similar neural representations) across training stages and mapping conditions. Numeral stimuli are represented in blue, and dot stimuli are represented in red. (Bottom) Multidimensional scaling (MDS) visualizations showing how symbolic (blue) and non-symbolic (red) number representations align across training stages.

Representational similarity analysis revealed that numerical distance effects - a hallmark of mature numerical cognition - emerged for symbolic representations by the end of mapping training (Figure 4, top panel, middle). This indicated rapid integration of symbolic numbers into a coherent representational structure. Multidimensional scaling demonstrated strong alignment between symbolic and nonsymbolic representations after cross-format comparison mapping, with correlation between representations increasing dramatically from 0.25 to 0.94 and normalized average distance decreasing from 0.53 to 0.12 (Figure 4, bottom panel, middle). This robust alignment provides a mechanistic explanation for the successful generalization observed.

#### Effect of symbolic specialization.

In Stage 3, we then examined the effects of continued training in the symbolic format alone. During symbolic training, the model maintained its generalization capacity but showed moderate forgetting of nonsymbolic skills, with accuracy declining from above 94% to around 75% (Figure 3, top panel). This forgetting was relatively evenly distributed across different numerical distances (Figure 3, bottom panel, right). Representational analysis revealed that symbolic training maintained high correlation between symbolic and nonsymbolic representations (0.96) but increased their average distance from 0.12 to 0.28 (Figure 4, bottom panel, left). This suggests that while ordinal relationships remained intact, the representational spaces began to diverge, explaining the selective forgetting observed.

### Generalization from cross-labeling

We conducted parallel analysis by altering Stage 2 to implement cross-label mapping to model the conventional educational approach where children learn to associate both symbols and nonsymbolic quantities with the same verbal labels (e.g., learning that both ”7” and seven objects are called ”seven”). This approach mirrors explicit instructional methods that rely on categorization and labeling rather than relational judgments, potentially creating separate representational pathways that converge on common output labels.

#### Limited generalization to symbolic comparison.

**Figure 5:**
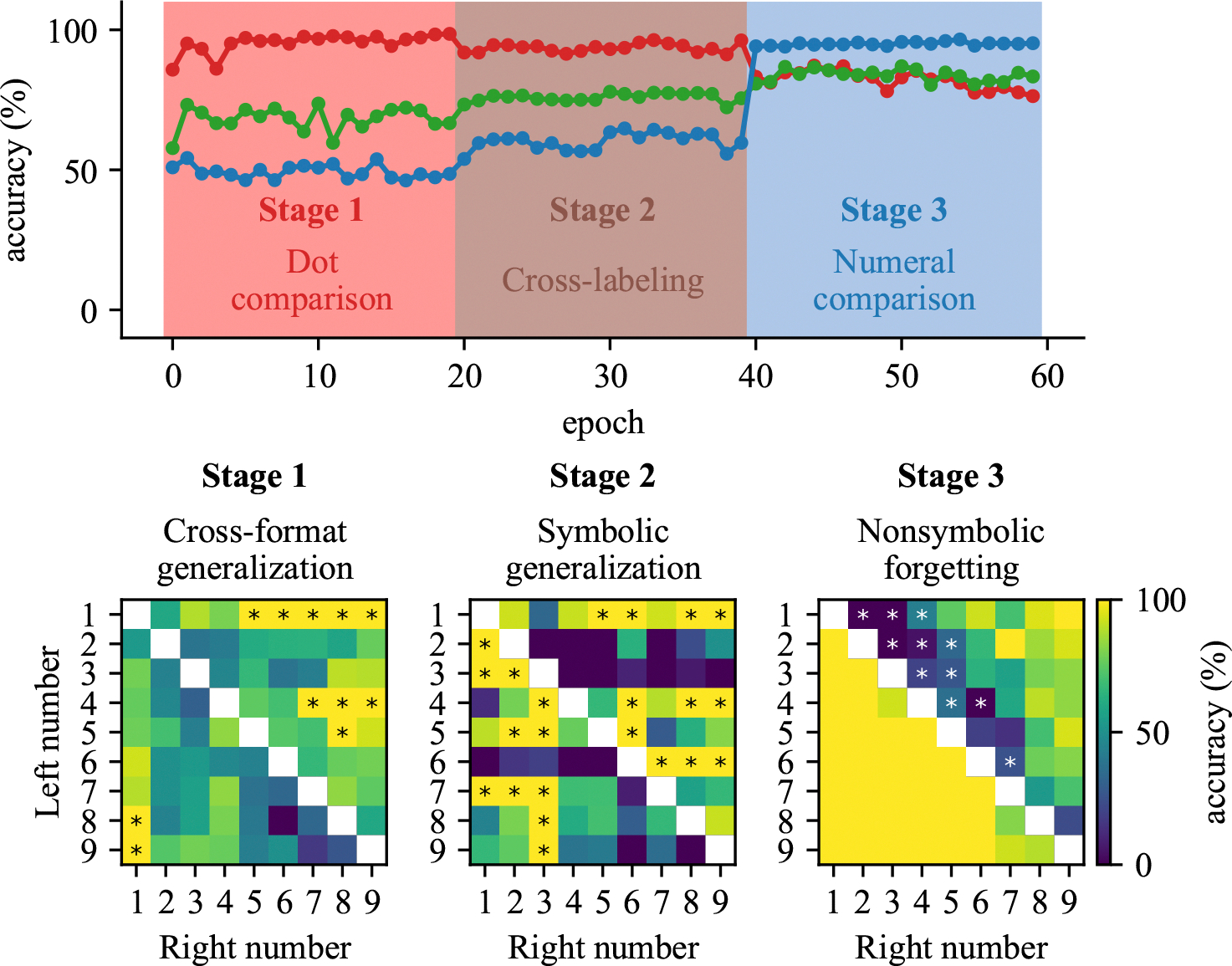
Performance of model developmentally trained with a cross-labeling training. (Top) Solid line represents the accuracy in test on the dot, cross-format, or numeral comparison task. The color of shaded area represents the new task that was trained. (Bottom) Pairs that are generalized and forgotten across training, from generalized cross-format pair in Stage 1, generalized symbolic pair in Stage 2, to forgotten nonsymbolic pair in Stage 3.

When mapping was established through cross-labeling (associating both formats with the same numerical labels), the model showed minimal generalization to symbolic comparison, achieving only 59% accuracy after mapping training despite 88% accuracy on its training task (Figure 5, top panel). Generalized symbolic pairs did not show a clear directional pattern, but scattered generalization across specific number pairs (Figure 5, bottom panel, middle).

#### Neural representational development.

**Figure 6:**
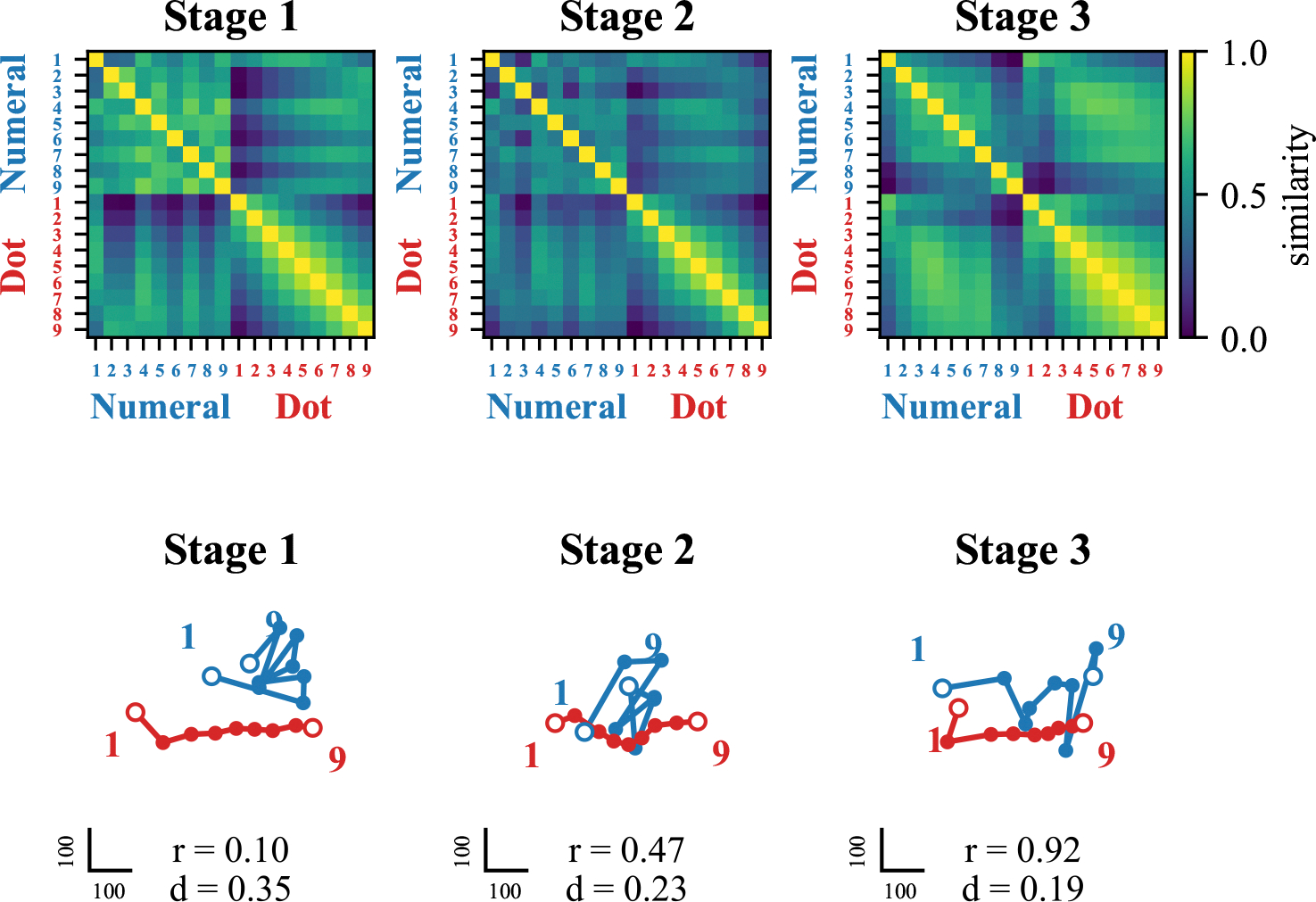
Progressive alignment of symbolic and non-symbolic number representation for model developmentally trained with a cross-labeling training. (Top) Neural representational similarity (NRS) matrices revealing distance effects (numbers closer in value have similar neural representations) across training stages and mapping conditions. Numeral stimuli are represented in blue, and dot stimuli in red. (Bottom) Multidimensional scaling (MDS) visualizations showing how symbolic (blue) and nonsymbolic (red) number representations align across training stages.

Symbolic numerical distance effects emerged only after explicit symbolic training in Stage 3, not during the mapping phase itself (Figure 6, top panel). This delayed emergence of structured symbolic representations indicates a weaker integration of numerical meaning during label-based mapping. Multidimensional scaling revealed limited alignment between symbolic and nonsymbolic representations after cross-labeling mapping, with correlation reaching only 0.47 and average distance remaining at 0.23 (Figure 6, bottom panel, middle). This weak alignment explains the poor generalization to symbolic comparison observed after mapping training.

#### Effect of symbolic specialization.

During symbolic training in Stage 3, the model improved its symbolic performance while demonstrating forgetting of nonsymbolic skills, similar to the comparison mapping condition (Figure 5, top panel). However, in this case, the pattern of forgotten pairs was concentrated on smaller numerical distances (Figure 5, bottom panel, right). Interestingly, the representational alignment actually improved during symbolic training, with the correlation increasing to 0.92 and the distance slightly decreasing to 0.19 (Figure 6, bottom panel, right). This suggests that explicit symbolic training can compensate for weaker initial mapping, although this improved alignment develops too late to support spontaneous generalization.

### Comparative analysis of mapping strategies

Direct comparison of the two mapping approaches reveals fundamental differences in how numerical representations develop. Cross-format comparison mapping produces earlier and stronger alignment between symbolic and nonsymbolic representations, facilitating robust generalization before explicit symbolic training. In contrast, cross-labeling mapping creates weaker initial alignment, requiring explicit symbolic training to achieve similar representational structure. Specifically, in Stage 2, representational alignment reaches a high correlation of 0.94 for cross-comparison but only 0.46 for cross-labeling, and distances of 0.12 for cross-comparison but only 0.23 for cross-labeling. The distinctive patterns observed across both conditions highlight representational alignment as the key mechanism underlying numerical semantic generalization. Strong alignment enables transfer of numerical meaning across formats, while weak alignment limits generalization despite successful task-specific learning.

## Discussion

Motivated by the developmental trajectory of numerical cognition in humans, we investigated how symbolic number representations emerge in artificial neural networks. We employed a systematic approach using comparison paradigms and mapping tasks to examine this process in detail. We trained a neural network through a developmental sequence, beginning with nonsymbolic number comparison, followed by one of two mapping approaches between symbolic and nonsymbolic representations, and concluding with symbolic number comparison training. The two mapping approaches we compared were: (1) a direct mapping through cross-labeling, where the model learned to associate both formats with common labels, and (2) an indirect mapping through cross-format comparison, where the model learned to compare numbers across formats. At each training stage, we tested the model with comparison tasks in dot, cross, and symbolic formats. Representational similarity analysis (RSA) (Kriegeskorte, 2008) and multidimensional scaling (MDS) (Mead, 1992) revealed that successful generalization from nonsymbolic to symbolic numerical processing coincided with the alignment of their neural representations in the model’s intraparietal sulcus layer, with stronger alignment producing more robust generalization across numerical formats.

Our findings revealed several key insights into numerical representation in neural networks. First, while both mapping conditions ultimately enabled accurate comparison of both symbolic and nonsymbolic numbers, only the indirect mapping approach (cross-format comparison) produced immediate generalization to purely symbolic comparisons. This unexpected finding suggests that cross-format comparison may create stronger representational linkages than shared labeling alone. Second, after completing the full training sequence, the models maintained above-chance performance on non-symbolic and cross-format comparisons, despite some decline in accuracy. This pattern mirrors developmental observations in humans, where early numerical skills persist even as symbolic proficiency increases.

Notably, our results showed a unique pattern of generalization during early training stages. As shown in Figure 3, the model demonstrated partial generalization to cross-format comparison tasks even before explicit mapping training, particularly for pairs containing the numeral 1. This early generalization to specific numerals suggests that some symbolic representations may be more readily integrated with their nonsymbolic counterparts, perhaps due to their distinctive visual features or frequency in training. This directional bias, absent in the cross-labeling mapping condition, bears striking resemblance to the SNARC (Spatial-Numerical Association of Response Codes) effect observed in human cognition, where numbers are mentally represented along a left-to-right spatial continuum. The emergence of this directional preference suggests that the cross-format comparison training may naturally induce spatial-numerical associations similar to human mental number lines, with smaller numbers mapped to the left and larger numbers to the right. The absence of this effect in the cross-labeling mapping condition indicates that comparison-based learning, which inherently focuses on relative magnitude relationships, may be particularly important for developing these spatial-numerical associations. Neural representational analyses yielded particularly compelling results, revealing the emergence of numerical distance effects—a hallmark of human numerical cognition—in both symbolic and nonsymbolic comparisons after training. These distance effects emerged at different training stages depending on the mapping condition, with crossformat comparison mapping producing distance effects for both symbolic and cross-format stimuli by the end of mapping training, while cross-labeling required symbolic training to achieve similar effects (Figures 4 and 6, top panels). Multidimensional scaling revealed that successful generalization coincided with structural alignment between symbolic and nonsymbolic representational spaces. Importantly, stronger alignment through indirect mapping corresponded with more robust generalization to symbolic tasks, while weaker alignment through direct mapping produced more limited generalization. The ”dot number line” and ”numeral number line” progressively aligned through training stages, with correlation values increasing from 0.25 to 0.94 in the cross-format comparison condition but only reaching 0.47 in the cross-labeling condition at the mapping stage (Figures 4 and 6, bottom panels). This quantitative difference in alignment strength directly corresponded to the difference in generalization performance. These findings extend previous work on representational alignment in rank comparison tasks (Nelli et al., 2023) to the domain of numerical cognition, demonstrating that similar principles apply across different cognitive domains. Critically, we show that this alignment can emerge through sequential learning of comparison tasks, suggesting it may be a fundamental mechanism supporting cross-domain generalization. The differential effectiveness of our mapping approaches has significant implications for educational interventions. Recent work with children experiencing mathematical learning difficulties has shown that training with indirect cross-format mapping not only normalized neural representations of numbers but also improved arithmetic problem-solving skills (Park et al., 2024). This suggests that the representational alignment we observed in our model may be a crucial mechanism supporting broader mathematical development. Our study opens several promising avenues for future research, including: (1) investigating whether children with mathematical learning disabilities show reduced alignment between symbolic and nonsymbolic number representations; (2) identifying specific neural mechanisms that might impair this alignment process; and (3) examining whether alignment-focused interventions can facilitate generalization to a broader range of mathematical skills beyond comparison tasks. In conclusion, our findings highlight representational alignment as a fundamental mechanism underlying the integration of different numerical formats and the emergence of numerical semantics in neural networks. This insight not only advances our theoretical understanding of numerical cognition but also offers promising directions for educational interventions to support mathematical development.
